# Order-Based Representation in Random Networks of Cortical Neurons

**DOI:** 10.1371/journal.pcbi.1000228

**Published:** 2008-11-21

**Authors:** Goded Shahaf, Danny Eytan, Asaf Gal, Einat Kermany, Vladimir Lyakhov, Christoph Zrenner, Shimon Marom

**Affiliations:** 1 Technion—Israel Institute of Technology, Haifa, Israel; 2 Hebrew University, Jerusalem, Israel; 3 University of Tübingen, Tübingen, Germany; University of Freiburg, Germany

## Abstract

The wide range of time scales involved in neural excitability and synaptic transmission might lead to ongoing change in the temporal structure of responses to recurring stimulus presentations on a trial-to-trial basis. This is probably the most severe biophysical constraint on putative time-based primitives of stimulus representation in neuronal networks. Here we show that in spontaneously developing large-scale random networks of cortical neurons in vitro the order in which neurons are recruited following each stimulus is a naturally emerging representation primitive that is invariant to significant temporal changes in spike times. With a relatively small number of randomly sampled neurons, the information about stimulus position is fully retrievable from the recruitment order. The effective connectivity that makes order-based representation invariant to time warping is characterized by the existence of stations through which activity is required to pass in order to propagate further into the network. This study uncovers a simple invariant in a noisy biological network in vitro; its applicability under in vivo constraints remains to be seen.

## Author Summary

The idea that sensory objects are represented by the order in which neurons are recruited in response to stimulus presentation was put forward over a decade ago, largely based on computational biology considerations. While intensively analyzed in simulation studies, the general biological applicability of this highly compacted and efficient representation scheme, as an ensemble neural code, was never established. In recent years, algorithmic and experimental technologies advanced to a stage that allows for facing the challenge; here we took advantage of this progress. We let a large-scale random network of cortical neurons develop on top of a microfabricated, multielectrode array that enables electronic interrogation of the network, stimulating through various points in space, and simultaneously recorded the resulting activities from a large number of neurons. We applied state-of-the-art classification algorithms and asked how well the rank order representation scheme handles categorization tasks. We show that recruitment order is generally applicable as an ensemble code; it emerges spontaneously in a large “structureless” network of neurons as a functional code that is invariant to significant temporal variance in spike times and spike rates and flawlessly classifies inputs on a trial-to-trial basis.

## Introduction

Sensory categorization is mediated, at least in part, by brain processes that extract information from the precise points in time at which neurons emit their first few spikes in response to the presentation of a sensory object [1]–[7]. In that context, a particularly attractive candidate representation primitive makes use of the order of neuronal recruitment, computed from the latencies to first spikes. The idea of representation by recruitment order is physiologically and computationally appealing because of its simplicity, rapidity, robustness and ease of implementation [7]–[10].

Spike order-based representation was shown to be applicable *in-vivo*, under conditions where the input has a fixed temporal order, either because of temporally structured stimulus features (e.g., [11]) or due to unique structure of peripheral receptor tuning curves [8]. Order based representation can also result from an underlying feed-forward network structure (e.g., [12]). But what if these constraints are relaxed? Is recruitment order applicable for representing stimuli that are not temporally ordered, in complex large-scale recurrent neural networks? If applicable, how does it handle trial-to-trial variations in spike times of individual neurons? How sensitive is it to the temporal resolution of ordering and the number of sampled neurons? How much of the network's classification capacity is conserved when absolute times of spikes evoked in response to a given stimulus are compacted to vectors of recruitment orders? The answers to these questions impact on the general applicability of recruitment order as an ensemble neural representation scheme.

Here we approach the above questions by examining the capacity of recruitment order to classify between multiple stimuli delivered to a large-scale recurrent network of cortical neurons that develops spontaneously *in-vitro*; this is an experimental model that matches the generic biophysical nature of the subject matter, and provides exquisite control of relevant variables. Key functional properties of *in-vivo* networks are conserved in this preparation [13], including cell types and their electrophysiological characteristics, synaptic and cellular level plasticity, developmental timeline and sensitivities to pharmacological agents. Sensory objects are defined in terms of identities of stimulating electrodes and the evoked neuronal activities are monitored through substrate-embedded array of spatially distributed extra-cellular recording electrodes.

We show that recruitment order reliably classifies input sources on a trial-to-trial basis and is invariant to significant temporal changes in absolute spike times of individual neurons. Classification accuracy monotonously increases with the number of sampled neurons, and steeply sensitive to the temporal resolution of spike ordering. The infrastructural origin of rank order representation is interpreted in terms of effective network topology.

## Results

### Time Warping of Cellular and Population Responses

For spike-timing based representation to be applicable, latencies between spikes should be consistent in repeated presentations of a given sensory object, and distinctive between different objects. Both requirements strongly depend upon trial-to-trial variability of spike times. While directly stimulated individual cortical neurons can respond very reliably in terms of spike latencies [14],[15], evidence for trial-to-trial variability in spike times and spike counts of network-embedded neurons *in-vivo* abound [16]–[21]. As shown below, a large-scale recurrent network of cortical neurons that develops spontaneously *in-vitro* presents a similar dichotomy: Latencies to first spikes in “receptive sheath neurons”—i.e., individual neurons that are directly activated by external stimuli—can be very reliable; in contrast, trial-to-trial variability of latencies and counts is extensive when “downstream” spikes are considered—i.e., spikes that are generated by propagation of the activity from the receptive sheath neurons deeper into the network. Note that the recurrent nature of the networks implies that a given neuron may, and in most cases does, serve in both groups.

We find that at stimulation frequencies of 3 Sec^−1^ or below, which is the range used in this study, the single spike evoked by each short (0.4 mSec) stimulus that is directly applied to a receptive sheath neuron, occurs within less then 6–7 milliseconds following the stimulus. In concurrence with previously reported measurements from cortical neurons *in-vitro* [22],[23], the latency from the stimulus to the response in that range of stimulation frequencies is very reliable (Figure S1).

**Figure 1:**
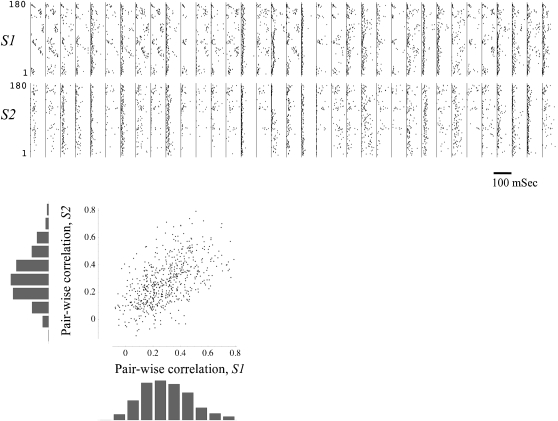
Latencies to first downstream spikes. Thirty-five columns are shown in the upper part of the figure, representing responses of 35 neurons (spike-sorted units) to two series of 180 stimuli that were applied at a rate of 0.2 Sec^−1^ each, first from one stimulation site (*S1*, top row) and then from another (*S2*, bottom row). In each column a vertical line depicts 10 mSec following stimulus time (see text), and a series of points represent the latencies to first spikes detected thereon in response to consecutive stimuli. Latencies greater then 100 mSec are not plotted (see scale bar). Bottom panel: Pair-wise correlation distributions for each of the two stimulation sources (bar charts). Each of the points in the main graph depicts the correlation between first spike latencies of a given pair of neurons, computed from responses to *S1* (X axis) and *S2* (Y axis); there are 595 points in the graph, representing all possible neuronal pairs.

In and by itself, reliable latency to first spike of directly activated neurons can support representation by recruitment order that is generated by stimulus dynamics (different receptive neurons activated at different times [8],[24]). But this is not what we are after here; the present study aims at the next level of processing, beyond the receptive sheath. Specifically, we ask how applicable recruitment order representation is *downstream* to the point of stimulus entry into a network, where spike time reliability is degraded by the dynamics of synapses, intricacies of propagation along axo-dendritic trees and the complexity of recurrent connectivity. Figure 1 shows latencies to first spikes measured downstream to the point of stimulus entry into a network. First spike latencies from 35 spike-sorted units [25] are shown, evoked in response to stimuli invading the network from two different well-defined loci (“sources”), *S1* and *S2*. To assure that we only look at downstream neurons (rather than receptive sheath neurons that are directly activated by the electrical stimulation), the first 10 mSec following each stimulus were removed from the data. Figure 1 (Top two panels) indicates that in spite of the relatively low rate of stimulation (compare with Figure S1), the latencies to first downstream spikes are severely warped over a range of 100 mSec and more, they wax and wane in a seemingly random, yet constrained manner. Note that the magnitude of time warping for a given neuron depends on the stimulation source. The bottom panel of Figure 1 shows that in many cases the correlation between latencies of different neurons is stimulus source specific.

**Figure 2:**
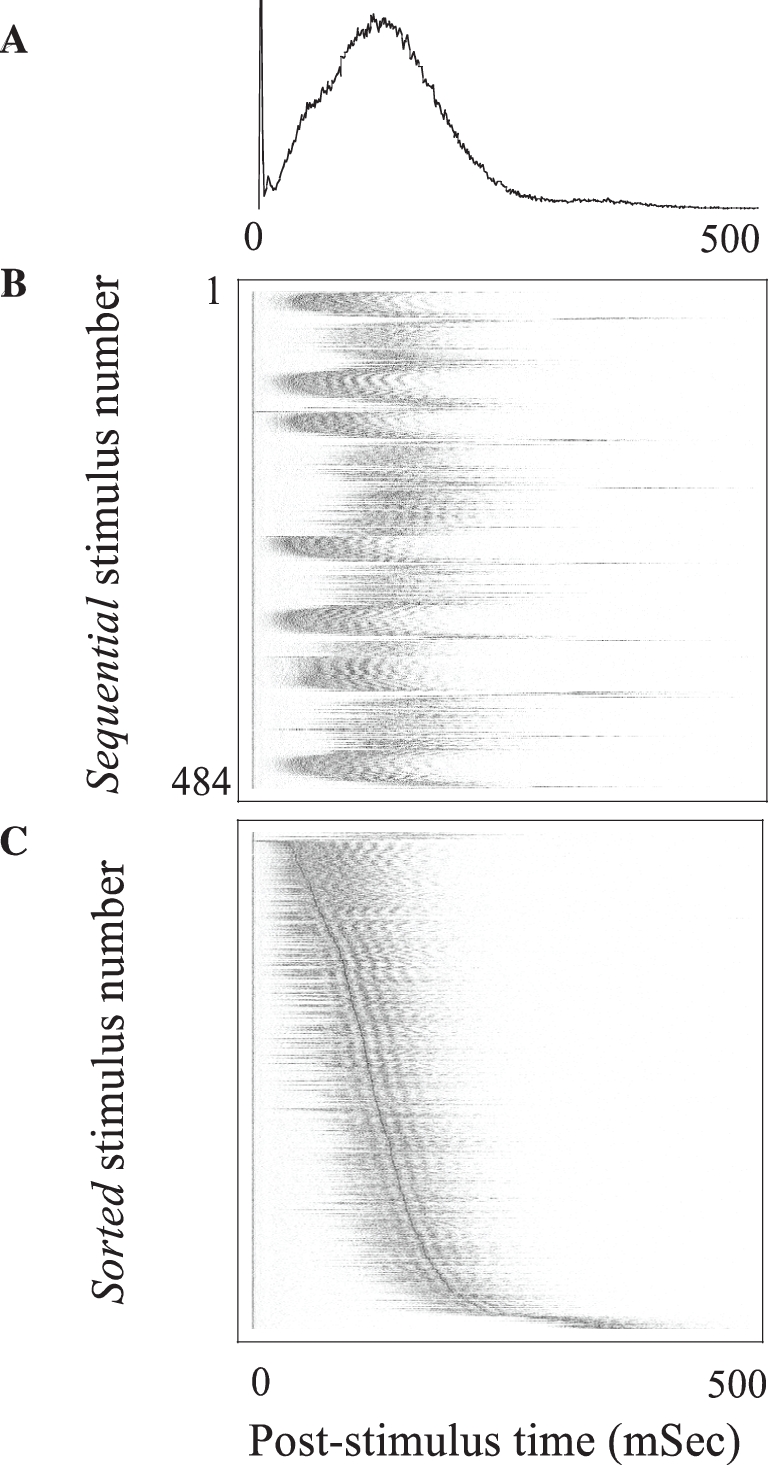
Latencies to population responses. (A) Population post-stimulus time histogram (pPSTH). 52 electrodes in which spikes were detected in >15% of the stimuli were considered for this analysis. The number of spikes recorded in a time window of 500 mSec following each of 484 stimuli was registered in 1 mSec time bins, averaged, normalized to peak and plotted in black line; the absolute value at the peak ∼100 mSec is ∼4 spikes/msec per 52 electrodes. The stimuli were applied from a single stimulation site at a frequency of 0.3 Sec^−1^. (B) Horizontal lines, coded by a grayscale in which maximal spike counts are depicted black, show the responses to each of the 484 individual stimuli. Note trial-to-trial variations. (C) The individual responses of panel 2B, sorted based on their time to peak. Note the range and multiplicity of time scales involved.

**Figure 3:**
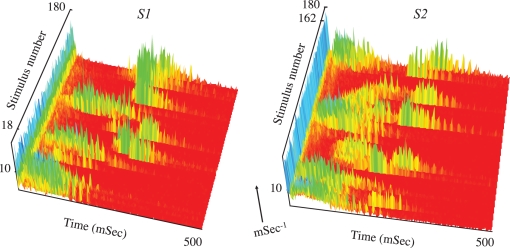
Network response to two different series stimuli, delivered from two separate sources (180 from each source), *S1* and *S2*. The stimuli were delivered intermittently at two different frequency (*f*) regimens: during the first 900 seconds the network was stimulated at frequencies *f_S1_* = 0.02, *f_S2_* = 0.2 Sec^−1^; in the following 900 seconds, the network was stimulated at frequencies *f_S1_* = 0.2, *f_S2_* = 0.02 Sec^−1^. In practice, for both regimens, every 10^th^ stimulus was applied from the “rare” stimulation source. Spike counts per mSec are coded by a grayscale in which maximal spike count is depicted white; note scale on the vertical axes.

The above time warping is also observed at the population level, provided that activity is not averaged across trials. The average network response, expressed in terms of a population post-stimulus-time-histogram (pPSTH), defined as the average number of spikes recorded throughout the network in a time window of 500 mSec following each stimulus, registered in 1 mSec time bins, shows a characteristic threshold-governed time amplitude trajectory that lasts 0.1–0.2 Seconds [26], comparable to numerous observations *in-vivo* [1], [27]–[29]. To differentiate from averaged population response, we denote the response of the network to a single stimulation event *network spike*, and define it in terms of the total number of neuronal action potentials counted over the entire population as a function of post-stimulus time [26]. Figure 2A and 2B show the pPSTH and the underlying variance between network spikes in response to a series of stimuli that were delivered to the network from a single stimulation site at a frequency of 0.3 Sec^−1^. Trial-to-trial variations appear in the time-delay between the stimulus and the peak of the network spike, as well as in the overall shape of network spikes. Note that the range of temporal variations extends over several tens of milliseconds within which the network response warps, shifts to the right (longer delays) and back to the left (shorter delays) in a graceful manner or, sometimes, in what seems like a sudden switch between response modes. To appreciate the multiplicity and range of time scales involved, we have sorted the data shown in Figure 2B based on the time-delays from the stimulus to the peak of each network spike (Figure 2C). Note the multiplicity of scales that are involved in the latency from stimulus to the peak and the width and the activity within network spikes, extending from below .01 Sec^−1^ up to the ∼40 Sec^−1^ gamma range. The range and overall nature of population time warping does not depend on the stimulation source. This is demonstrated in Figure 3 that shows time warping of network spikes in response to two series of stimuli, delivered from two different stimulation sites, *S1* and *S2*.

The above non-monotonic changes in absolute time delays between stimulus and neuronal responses at the levels of individual neurons and neuronal populations, set constrains on the capacity of temporal measures to reliably classify input sources on a trial-to-trial basis. In what follows we show how representation by recruitment order, computed from the latencies to first spikes, handles time-warped neuronal responses.

### Stimulus-Specific Recruitment Orders

For a propagation path (and hence for a recruitment order) to be invariant to neuronal response time warping in a large scale recurrent network, one of the following two options must be fulfilled: (*i*) Dynamics of membrane variables and synaptic efficacies are scaled and homogeneously distributed throughout the network; the idea is that under such conditions, paths of less resistance to propagation of activity remain stable. This option, however, is difficult to conceive biophysically and incompatible with previously reported results (e.g., [30]). (*ii*) Propagation paths, and hence order of recruitment, are constrained by chains of neuronal stations through which activity is required to pass in order to propagate further into the network, regardless of the status of membrane and synaptic dynamics; such stations are natural consequences of physical or effective connectivity that are inherent to the concept of synfire-chain [12],[31] or certain forms of broadly-distributed network connectivity [26],[32]).

**Figure 4:**
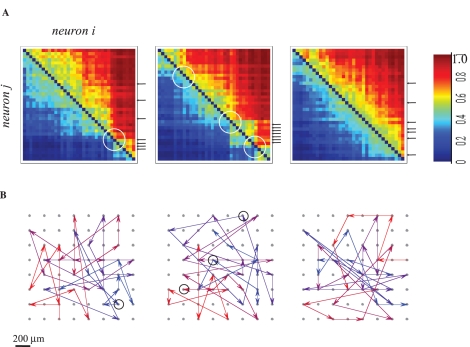
Demonstration of neuronal stations through which activity is required to pass in order to propagate further into the network. (A) Pair-order probability matrices, generated from responses to three different stimulation sources of one network: The matrices (each for one of the three stimulation sources) depict the probability (color-coded) of each neuron to precede every other neuron. Neurons are presented in these matrices sorted by their average rank. White circles depict the impact of presumed bottlenecks outside the sampled area. Small black arrows to the right of the middle panel depict a cluster of neurons that tend to respond close to each other in terms of their recruitment order; each arrow indicates one of these neurons. The dispersion of these arrows in the other (right and left) panels indicates that the rank of any given neuron is stimulus site specific. (B) Activation pathways for the three sources shown in (A) above. The average rank vector of the responses to each source is projected onto a map of the physical locations of each electrode. Note that propagation lines that connect between electrodes that are horizontally or vertically aligned might mask each other and give the impression that a sequence has more than one endpoint; to overcome this graphical problem, a color-coding for the rank of each arrow (Red to Blue) is used. Circles depict physical locations of neurons circled in 4*A*.

To identify chains of neuronal stations in large-scale neuronal networks under time-warping conditions, we have analyzed the recruitment order relationship between all recorded neuronal pairs: Given *n* neurons, there are *n*(*n*−1) different (*i*, *j*) neuronal pairs (*i* = 1, 2, 3, … *n*; *j* = 1, 2, 3, … *n*). The first spike times of a pair (*i*, *j*), in response to each stimulation event, may appear in one of two orders, *i→j* and *j→i* (we disregard the possibility of complete synchronous occurrence, for the sake of simplicity). We measure the probability of *j* to precede *i* for all possible pairs. If a given neuron (or a group of neurons) is an ideal station through which activity is required to pass in order to propagate further into the network, we expect that it always be preceded by activity of a given subset of neurons, and always be followed by the complement set of neurons. In other words, each genuine station in a chain would act like a bottleneck, separating all other neurons (or groups of neurons) into two groups based on the temporal relation between their first spikes and its own: a group of preceding neurons and a group of following neurons. Figure 4A demonstrates three pair-order matrices, generated from responses to three different stimulation sources of one network: The matrices (each for one of the three stimulation sources) depict the probability of each neuron to precede every other neuron. Neurons are presented, in each of the matrices, sorted by their average rank. All three matrices clearly show that recruitment is ordered (see Figure S2 for more examples from different networks). The left and middle matrices of Figure 4A demonstrate cases in which our electrodes picked a small number of clear clusters of neurons; the right matrix demonstrates a fairly homogeneous arrangement along the diagonal and no clear clusters of neurons. The variety of matrix forms shown in Figure 4A (and Figure S2) probably reflects the effect of sparse spatial sampling of a common underlying structure that enforces ordered recruitment: a chain of neural stations. Consider the simple three-stations arrangement, X→Y→Z. Let us assume that X, Y and Z are clusters of highly interconnected neurons, and that there is some overlap between these clusters (i.e., some neurons in cluster X are also part of cluster Y and so on). Cluster X activates cluster Y, which, in turn, activates cluster Z. In that respect, Y is a bottleneck station between X and Z. If cluster Y is *outside* the electrodes sampling area, the pair-order probability matrix is expected to show sharp separation between clusters, X and Z; the left and middle panels show this type of behavior, where white circles indicate the existence of bottlenecks (the equivalents of Y) that reside outside the sampling area. On the other hand, when all (or most) of the clusters are within the sampled area, we expect to see a more homogeneous diagonal arrangement (i.e. a chain of bottleneck stations) like the one exemplified in the right-hand panel of Figure 4A.

Figure 4A also tells us that the rank order of different neurons is stimulus site specific: The small black arrows to the right of the middle panel depict a cluster of neurons that tend to respond close to each other in terms of their recruitment order; each arrow indicates one of these neurons. The dispersion of these arrows in the other (right and left) panels indicates that the rank of any given neuron is stimulus site specific. Thus, neurons appear at different ranks in responses to the three different stimulation sites, providing the infrastructure for recruitment order classification of input sources. Figure 4B shows that the three paths shown in Figure 4A do not result from a spatial wave-like propagation of activity across the recording area; rather, propagation paths appear to randomly connect between the recording electrodes, suggesting that the underlying structures are embedded in a non-trivial manner in space.

In what follows we show that based on the two key features demonstrated in Figure 4A, (*ordered recruitment*, which is *stimulus specific*), rapid and reliable classification of inputs is readily obtained by use of unsupervised as well as supervised algorithms.

### Unsupervised Stimulus Classification by Recruitment Order on a Trial-to-Trial Basis

Throughout this study, we have imposed two constraints on which spikes are considered for analyses; both constraints are meant to avoid trivialization of the order-based classification task: (*i*) we omitted spikes that were evoked up to 10 mSec following each stimulus (i.e. latency 0 means 10 mSec following a stimulus), thus avoiding classification by first spike latencies emitted from receptive sheath neurons. (*ii*) We only considered first spike latencies in neurons that responded to more than 90% of stimuli from *all* sources, thus making sure that classification is not based on neuronal identities (in which case the task is trivialized by relying on a neuron, or group of neurons, that has high response probabilities to stimuli delivered from one of the sources, but not from other sources).

**Figure 5:**
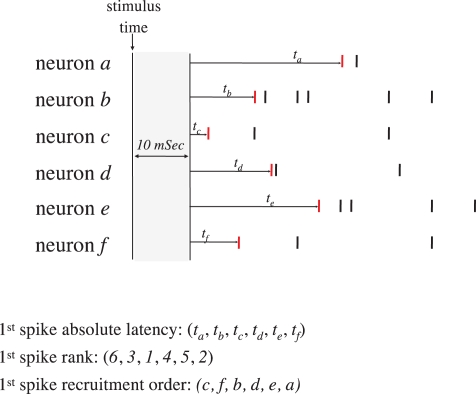
An illustration of data reduction and terminology. Picture 6 neurons (*a–f*) responding to a single stimulation event by evoked spikes (vertical lines). Spikes emitted during the first 10 mSec post stimulus are ignored. The recruitment order is derived from latencies to first spikes (Red vertical lines). Neurons that fired within the same time bin were either ranked according to their alphabet (for string metric analyses) or credited an equal rank (for SVM analyses; see Materials and Methods).

Figure 5 demonstrates the process of extracting recruitment order from a network response to a stimulus. Note that recruitment order is a reduced form of absolute latencies to first spikes, a fact that will become crucial in a later section of this report, where we address the question of how much of the network capacity to classify input sources is lost in this reduction.

To test the capability to classify inputs by recruitment order, we start by applying an unsupervised classification algorithm, that is – classification without the need to learn from labeled examples. To that end an order metric was applied, such that the distance between different recruitment orders can be measured: A single character symbolizes each neuron, and words are obtained, each of which represents the first spike order of neuronal recruitment in response to a given stimulus. For example, the word cgbdhefa stands for the order in which 8 neurons (a–h) were recruited in response to a given stimulus. The word cagbdhief stands for a response to another stimulus (from the same or different input source), but this time 9 neurons (a–i) were recruited to respond. The Levenshtein Edit Distance string metric was used for measuring the distance between any two strings, expressed in terms of the minimum number of editing operations (insertion, deletion, or substitution) needed to transform one string into the other.

**Figure 6:**
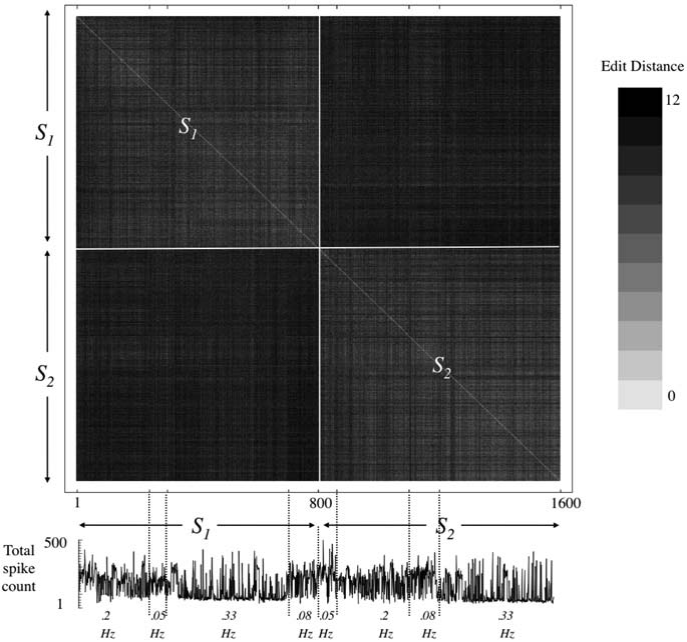
Order-based representation. Bottom trace: Stimuli were applied intermittently from the two sites, at four different frequency regimens (*f*, Sec^−1^): *f_S1_* = 0.2, *f_S2_* = 0.05; *f_S1_* = 0.05, *f_S2_* = 0.2; *f_S1_* = 0.33, *f_S2_* = 0.08; and *f_S1_* = 0.08, *f_S2_* = 0.33. The total number of spikes detected in all the electrodes within 500 mSec following each of the 1600 stimuli is sorted according to stimulus site (S1 and S2), the order in which stimuli were applied, and the frequency of stimulation. Note that the four different frequency regimens yielded very different spike counts. Upper panel: A matrix of Edit Distances between first spike recruitment orders evoked in response to the repeated stimuli applied from the two stimulation sites. The matrix is temporally aligned to the bottom panel. Distances are computed using the Levenshtein Edit Distance. Clusters of similarity that match the two sources (*S1* and *S2*) are clearly observed (depicted).

Figure 6 demonstrates classification between two input sources based on the Edit Distance metric. A network was stimulated from two sources (*S1* and *S2*) intermittently, at four different frequency regimens. The top panel of Figure 6 shows the resulting Edit Distance matrix; responses are ordered according to their stimulation source, revealing clusters of similarity that clearly match the two sources of input (depicted by white lines). Note that the four different frequency (*f*) regimens yielded very different population spike counts (low panel of Figure 6). Yet, the representation by recruitment order remains invariant to these changes. In nine different networks that were challenged with a two-source classification task, cluster analyses (standard hierarchical algorithm, forced to identify two clusters) using Edit Distance metric yielded average classification accuracies ranging from 0.6 to 0.98 (median = 0.72; SD = 0.13). The arbitrariness of our choice of Edit Distance metric is acknowledged; to avoid possible bias in the interpretation of our results, other order-based metrics were applied (e.g. Spearman correlation and Euclidian metrics), yielding qualitatively similar results (data not shown).

### The Compression of Precise Latencies to Recruitment Orders Does Not Degrade Classification Accuracy

The electrical activity of neuronal networks is expressed in terms of neuronal identities and their absolute spiking times; recruitment order is a dramatically reduced form of that data. How much of the classification capacity is conserved when absolute first spike times, which are evoked in response to a given stimulus, are compacted to lists of recruitment orders? To answer that question, an estimate for classification capacity should be obtained for both absolute spike times and recruitment order representations. The dimensionality and statistical properties of our experimental data renders simple and direct estimation of classification capacity from response distributions impossible. To circumvent this limitation an alternative approach is adopted, where a lower bound on the classification capacity is estimated by the performance of a general purpose supervised classifier, trained to recognize the sources of stimulation. A Support Vector Machine (SVM) with an adaptive Gaussian kernel (see Materials and Methods for details) was trained to classify the different input sources based on labeled examples of the data, and the classification capacity was estimated by its performance on a test data set. In nine different networks that were challenged with a two-source classification task, the median accuracy (test sets only) obtained by use of absolute time to first spikes at 1 mSec resolution was .99 (SD = 0.03); the median accuracy obtained by use of recruitment rank order using 1 mSec resolution is .98 (SD = 0.12). In six different networks that were challenged with a five-source classification task (data shown in the context of the subsequent section), the median accuracy (test sets only) obtained by use of absolute time to first spikes at 1 mSec resolution was .91 (SD = 0.03); the median accuracy obtained by use of recruitment rank order using 1 mSec resolution is .94 (SD = 0.04). (Note that the slightly better performance of the recruitment order based classifier, compared to absolute time based classifier, reflects the lower dimensionality of the first relative to the latter, and the entailed effect on the sample size required for learning.) Thus, in a two-source and five-source classification tasks, reduction from spike latencies to recruitment order representation is practically lossless. The classification capacity of these networks in a six input sources task yields similar results (data shown in the context of the subsequent section).

### Sensitivity of Order-Based Classification to the Number of Sampled Neurons and Temporal Resolution

**Figure 7:**
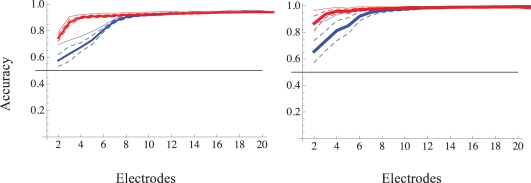
Sensitivity of classification accuracy to the number of sampled electrodes. Two examples from two different networks are shown (left and right panels). In both cases the networks were challenged with a two-stimulation sites classification task (black horizontal lines depict chance accuracy level). Support vector machine (SVM) algorithm with a Gaussian Radial Based function kernel was applied to vectors of training sets (see Materials and Methods). The resulting classifiers, one for first spike absolute latency and the other for recruitment order, were then validated using test sets vectors. Mean test accuracy (thick lines) and confidence intervals (interrupted lines) are calculated from 200 random combinations of electrodes per given sample size from all analyzed electrodes. Classification accuracy of first spike latency is depicted Red; that of recruitment order is depicted Blue. Thin lines depict the best classifier for each group size.

Up to this point, the presented classification results were based on all the electrodes that responded beyond the 90% criterion mentioned above. Theoretically, *n* neurons provide a space of *n*! possible representations; thus, for instance, six sources can, in principle, be classified based on the recruitment order of only three neurons. How sensitive is classification by recruitment order to the number of sampled neurons? Figure 7 shows the rank order representation accuracy as a function of the number of sampled electrodes for two different networks (two source discrimination task). Here, each point in a continuous line depicts the mean accuracy of 200 random combinations of *n* electrodes (abscissa). Note the monotonic increase in accuracy as a function of the number of sampled electrodes; similar results were obtained in six experiments of five sources classification task, shown in Figure S3, left panel. Moreover, even though the classification accuracy based on spike timing is significantly higher than that of rank based representation at very small numbers of sampled electrodes, this difference disappears when the number of electrodes increased. In other words, as the number of sampled neurons increases, the information carried by spike times becomes redundant to the information carried by recruitment order. The apparent difference between the performances of the two classifiers in low electrode numbers implies that absolute spike times carry information about the stimulus source that is not captured by recruitment order. It is important to note, however, that the information carried by the exact latencies does not mean that this information is available to decoders that are based on precise times (e.g. coincidence detectors). In fact, our data implies the contrary: The time-warping exemplified in the first part of the results suggests that absolute times to first spikes are not precise, at least in this preparation, and some non-trivial transformation of spike times is required before a satisfactory classification may be achieved. The relative simplicity of the recruitment order over spike times coding is apparent in the success of classification using unsupervised methods (Figure 6); this method was unsuccessful when applied to spike times (various distance metrics were tried; data not shown). Indeed, it seems likely that a tradeoff exists between the number of sampled neurons and the complexity of the neural code.

**Figure 8:**
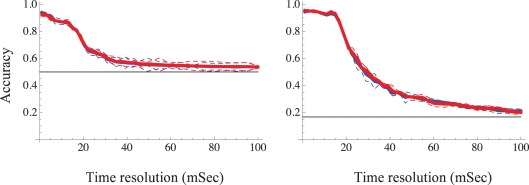
Sensitivity of classification accuracy to the temporal resolution of sampled latencies to first spike times. Two examples from two different networks are shown (left and right panels). In the left panel a network that was challenged with a two stimulation sites classification task is shown; the right panel shows a case of a network that was challenged with a six stimulation sites classification task (black horizontal lines depict chance level). Support vector machine (SVM) algorithm was applied (see Materials and Methods and caption of Figure 7). Mean test accuracy (thick lines) and confidence intervals (interrupted lines) are calculated from a 5-fold cross validation procedure per given time resolution. Classification accuracy of first spike latency is depicted Red; that of recruitment order is depicted Blue.

Figure 8 demonstrates the impact of temporal resolution on the accuracy of classification in a case of two sources classification task (left panel) and six sources classification task (right panel). Data was binned at temporal windows ranging from 1–100 mSec, and latency and rank vectors were prepared as explained above (Figure 5). Figure 8 shows that, in general, the classification capacity is very high as long as the temporal resolution is in the range of 10 mSec or less, decreasing significantly at lower resolutions; similar results were obtained in six experiments of five sources classification task, shown in Figure S3, right panel. Importantly, the reduction of spike time data (Red) to recruitment order (Blue) does not degrade the classification accuracy throughout the range of analyzed resolutions. This result is evident in all of our experiments (data not shown). In the nine different networks that were challenged with a two-source classification task, a 50% drop in accuracy was observed at a median temporal resolution of 43 mSec (±33 SD; range 15–71 mSec). The issue of temporal resolution required for accurate representation by recruitment order is rather subtle: our data indicates that while timing of individual spikes is lost in the transition to recruitment order, the temporal precision required for comparing the relative spike timing between different neurons is crucial for an accurate classification.

## Discussion

The extensive [33] and often conflicting array of hypotheses concerning neuronal representation of objects (sensory, motor or “internally generated” ideas or concepts), reflects indeterminacy of data to theory. Regardless of what the nature of neural representation turns out to be, it should conform to elementary physiological constraints. Perhaps the most severe constraint in that respect is the multiplicity and wide range of timescales that are characteristic of neuronal excitability and synaptic communication: At each and every level of observation, physiologists report an ever-increasing range of reaction time scales that are involved in the generation of action potentials and their transformation to post synaptic signals [34]–[38]. It is hardly surprising, therefore, that the temporal structure of neuronal responses to repeated presentations of stimuli becomes inherently warped on a trial-by-trial base, reflecting the long tail of sensitivities to previous activation histories mediated by activity-dependent reactions underlying both exciting and restoring forces. Here we show that the effective structure that emerges spontaneously in large random networks of cortical neurons leads to representation by recruitment order that is invariant to response time warping. We bring evidence for the existence of neuronal stations through which activity is required to pass in order to propagate further into the network. We find it convenient to think about these sequences of neuronal stations in terms of chain-like effective structures [12]; thus, even in the face of activity-dependent changes in synaptic efficacies or membrane excitability, activity has nowhere else to go but through ordered stations, reassuring that the rank remains stable. Network architecture, in that sense, serves to protect the representation by order from the effects of the dynamics driven by activity dependence of reaction rates. Taken together with the observation that the ranking is stimulus site specific, the basic conditions for application of recruitment order representation are satisfied. (Video S1 demonstrates a functional implementation of the above results: a Braitenberg vehicle that classifies objects in its visual field based on neuronal activity in a large-scale biological neural network. See caption of Video S1 for methodological comments).

While ordered patterns of activations are observed at various spatiotemporal scales *in-vivo* and *in-vitro* in several neuronal preparations (e.g., [8], [11], [26], [31], [39]–[42]), the general applicability of representation by recruitment order at the random ensemble level is demonstrated, for the first time, in the present study. We provide a direct measure for the efficacy of rank order representation in actual classification tasks under well-controlled experimental conditions in large-scale recurrent networks of cortical neurons. Recruitment order is highly sensitive to the spatial features of stimuli and accurately classifies them on a trial-to-trial basis. The accuracy of spatial representation by the order of neuronal recruitment monotonically increases with the number of sampled neurons, and decreases with ordering time resolution. Furthermore, we show that the process of data compression, from absolute first spike latencies to recruitment order, is lossless from the point of view of stimulus classification accuracy. These results, even when taken together with the simplicity, rapidity, robustness and ease of physiological implementation of representation by recruitment order do not necessarily imply that it is superior to other representation primitives (e.g. rate based or precise time delays); more likely the balance between these features and task constraints govern the usefulness of a representation primitive in a given context.

Several ways were proposed to realize a biologically plausible mechanism to decipher recruitment order. These include, for instance, a simple feed-forward network with shunting inhibition [7], a decoder that is based on spike timing dependent plasticity that re-distributes synaptic weights at the single neuron level [43], as well as a tempotron-based decoder that relies on adaptive integration time mechanisms [44]. Another issue that relates to the physiological plausibility of a decoder for recruitment order, has to do with the temporal reference point to which the code is time locked: In our experimental design, the classification is based on prior knowledge of the time at which a stimulus is delivered, but under real life situation such information is not available for the decoder. Recent studies, however, show that the temporal reference issue, which comes up whenever a time-based representation scheme is proposed, can be handled either by temporally referencing to population activity onset [45], or by relying on time-based synaptic plasticity processes that tune the distribution of synaptic weights such that a neuron becomes sensitized to early spikes in a pattern [43].

Finally, from a more general perspective, it is interesting to contemplate on the possible functional relations between activity dependence of molecular transition rates underlying neuronal excitability (e.g., [34]), the space of possible combinations of precise latencies to first spikes and its degeneration to the form of recruitment order: While activity-dependence of neuronal reactions is a valuable driving force for exploration in a variety of adaptation and learning processes (where representations are modified), it must be balanced by mechanisms that allow for stabilization and hence exploitation of existing representations. Representation by recruitment order provides a particularly attractive solution to this tradeoff: neurons dynamically change their absolute spike times relative to a reference signal (e.g. stimulation time), thus exploring the space of possible associations driven by machineries of spike-timing dependent plasticity (e.g., [46],[47]). Since many combinations of latencies to first spikes may realize any given representation by recruitment order, existing representations are invariant to the exploration process, as long as the latter does not degrade the order of neuronal recruitment. Effectively, a separation is formed between the level of absolute time delays, where exploration for new representations occurs, and the level of recruitment order where representations are stable enough to adaptively interact with the environment. The analogy to the separation between mutations at the genomic level, and selection at the proteomic (phenotypic) level immediately comes to mind.

## Materials and Methods

### Network Preparation

Cortical neurons were obtained from newborn rats (Sprague-Dawley) within 24 hours after birth using mechanical and enzymatic procedures described in earlier studies [13], [22], [26], [30], [48]–[50]. The neurons were plated directly onto substrate-integrated multi-electrode arrays and allowed to develop functional and structural mature networks over a time period of 2–3 weeks. The number of neurons in a typical network is in the order of 300,000, over an area of ∼300 mm^2^; various estimates of connectivity suggest that each neuron receives ∼1000 synapses, with ∼10% of these synapses being inhibitory (see [13] for a comprehensive review of the preparation). The preparations were bathed in MEM supplemented with heat-inactivated horse serum (5%), glutamine (0.5 mM), glucose (20 mM), and gentamycin (10 µg/ml), and maintained in an atmosphere of 37°C, 5% CO_2_ and 95% air in an incubator as well as during the recording phases. Multi electrode arrays (MEAs) of 60 Ti/Au/TiN electrodes, 30 µm in diameter, and spaced 200 µm or 500 µm from each other (Multi Channel Systems, MCS, Reutlingen, Germany) were used. The insulation layer (silicon nitride) was pre-treated with poly-d-lysine. Long experiments lasting over 3 hours were conducted using a slow perfusion system with perfusion rates of ∼100 µL/hour.

### Measurements and Stimulation

A commercial 60-channel amplifier (MEA-1060-BC, MCS, Reutlingen, Germany) with frequency limits of 1–5000 Hz and a gain of ×1024 was used. The MEA-1060-BC was connected to MCPPlus variable gain filter amplifiers (Alpha-Omega, Nazareth, Israel) for further amplification. Rectangular 200 µSec biphasic 10–50 µA current stimulation through randomly chosen pairs of adjacent MEA electrodes was performed using a dedicated stimulus generator (MCS, Reutlingen, Germany) coupled to a blanking circuit that disconnects the amplifiers during each input pulse. Data was digitized using two parallel 5200a/526 A/D boards (Microstar Laboratories, WA, USA). Each channel was sampled at a frequency of 16–24 ksample/second and prepared for analysis using either the AlphaMap interface (Alpha Omega, Nazareth, Israel) or a dedicated Matlab (MathWorks, Natwick, MA, USA) interface developed by two of the authors (D.E. and C.Z.). Thresholds (×8 RMS units; typically in the range of 10–20 µVolt) were defined separately for each of the recording channels prior to the beginning of the experiment. All the activity recorded in the 60 electrodes up to 500 mSec following each stimulus were collected and stored for analyses. Where indicated, spike sorting procedures were applied, using the AlphaSort PCA package (Alpha-Omega, Nazareth, Israel). Previous studies [13], [22], [26], [30], [48]–[50] show that the rate of spontaneous activity in these networks is, at least, one order of magnitude smaller compared to the activity evoked by stimulation, both when considered at the level of individual neurons as well as at the level of population responses; the interference of spontaneous activity with our analyses and interpretation of the results is minute.

### Stimulus Classification Experiments

Mature networks were chosen for experimentation based on their ability to reliably respond to more than one source of low frequency (0.05 Sec^−1^) stimulation. Reliability of response is defined as a reverberating network activity (that is time locked to a stimulus), observed in over 50% of stimulus presentations. The classification results presented here are based on a data set from 15 networks that were exposed to two (*n* = 5), three (*n* = 2), five (*n* = 6) and six (*n* = 2) different stimulation sources. In the two-source classification tasks the stimuli were delivered at several frequencies as explained in the results section. In all cases, the order at which stimuli were delivered through different stimulation sources was shuffled throughout the experiment.

### Classifiers

Wolfram's Mathematica 5.2 environment was used for calculation of the Levenshtein string metric and for cluster analysis. Neurons that fired within the same time bin were ranked according to their alphabet. SVM classification analysis was performed using MCSVM_1.0 (http://www.cis.upenn.edu/~crammer), a *C* code package for multi-class SVM [51] using Gaussian Radial Based Function kernel. Kernel parameter and confidence intervals were set by a 5 fold cross-validation procedure. For SVM analyses, neurons that fired within the same time bin were credited an equal rank.

## Supporting Information

Figure S1 Dynamics of first spike latencies in pharmacologically isolated neurons. Excitatory synaptic transmission is blocked by application of 50 µM APV and 10 µM CNQX. As a result, all spontaneous activity and stimulus evoked reverberating responses were completely abolished. The network was then stimulated through all 60 electrodes sequentially to locate stimulation sites that effectively evoke direct, electrically induced action potentials. After selection of stimulating electrodes each network was stimulated by a short pulse (400 µSec) for 100 seconds (ordinate) at three different frequencies (0.3, 3 and 30/Sec; Green, Black and Red, respectively) in a shuffled manner. Five minutes were allowed for recovery between each stimulation series. Examples from five neurons are shown. The abscissa shows time post stimulus, in mSec. Failures to respond are plotted at time 0. Under these experimental conditions, neuronal responses to above-threshold stimuli reflect direct generation of action potentials in neurons that are nearby stimulating electrodes, free from the dynamics of synaptic transmission. At frequencies of 3/Sec or below, neurons are very reliable in their responses to directly delivered stimuli. At the higher frequency regimen, response jitter develops gradually over several seconds.
(4.6 MB PDF)

Figure S2 Pair-order probability matrices. More examples of pair-order probability matrices, similar to those shown in Figure 4A. Each boxed set of matrices comes from a different network. Each of the matrices within a box is constructed from responses to a different stimulation source (networks that were exposed to two and three input sources are shown). Numbers to the left of each box depict preparation (network) identity. All cases show ordered recruitment; in most cases matrices of different stimulation sources (within each box) look different. Indeed, the statistical analyses shown in Figures 6, 7, 8 and Supplementary Item 3 clearly indicate that these differences are significant and allow for rapid and reliable classification of inputs by use of unsupervised as well as supervised classifiers (15 networks tested altogether). Note: Neurons that are less “committed” to their average rank appear as horizontal (and vertical) smeared lines throughout the matrix.
(4.6 MB PDF)

Figure S3 Examples of five-sources classification task. Results from six different networks that were challenged with a five-sources classification task. While variance in the sensitivities of different networks to both the number of sampled neurons and temporal resolution is apparent, the overall picture is fairly robust. Left: Sensitivity of classification accuracy to the number of sampled electrodes was measured in a five-stimulation sites classification task. Horizontal line depicts chance accuracy level. Support vector machine (SVM) algorithm with a Gaussian Radial Based function kernel was applied to vectors of training sets (see methods). The resulting classifiers were then validated using test sets vectors. Mean test accuracy and standard deviation are calculated from 10 random combinations of electrodes per given sample size from all analyzed electrodes, using a 10-fold cross validation procedure. Classification accuracy of first spike latency is depicted Red; that of recruitment order is depicted Blue. Right: Sensitivity of classification accuracy to to the temporal resolution of sampled latencies to first spike times was measured in a five-stimulation sites classification task. Horizontal line depicts chance accuracy level. Support vector machine (SVM) algorithm was applied (see methods and caption of Figure 7). Mean test accuracy and standard deviation are calculated from a 30-fold cross validation procedure per given time resolution. Classification accuracy of first spike latency is depicted Red; that of recruitment order is depicted Blue; adjacent Red-Blue curves result from the same experiment (network).
(4.6 MB PDF)

Video S1 Braitenberg Vehicle. In a small yet seminal book titled Vehicles: Experiments in Synthetic Psychology (MIT Press, 1986), Valentino Braitenberg describes a set of thought experiments in which agents with simple structure behave in human-like ways; Braitenberg blatantly put forward the hypothesis that the primitives for realizing such machines are cellular and synaptic processes that are amenable for physiological characterization. The reasoning and results presented in this study make the realization of a Braitenberg vehicle that classifies objects in its visual field using a large-scale network of biological neurons a trivial matter. This is demonstrated in the attached clip that was prepared by Danny Eytan, David Ben Shimol and Lior Lev-Tov from the Technion - Israel Institute of Technology. The main text and data of the present study shows that the physical loci from which stimuli are delivered to a recurrent, large scale random network of cortical neurons, albeit causing temporally “noisy” neuronal responses, may be fully classified using the temporal order at which neurons are recruited by the different stimuli. Here, an application of this idea, in the form of a Braitenberg vehicle, is demonstrated: Inputs from the two (Right and Left) ultrasonic “eyes” of a Lego Mindstorms vehicle are sampled at 0.2 Hz and translated into stimulation of a large random network of cortical neurons at two different sites. The side corresponding to the nearest visual object (relative to vehicle's longitudinal axis, depicted by a red arrow) is classified using an Edit-distance metric based on the recruitment order of 8 neurons, similar to procedures shown in Figure 6 of the manuscript. Based on the classified activity, a command is sent to the appropriate motor attached to one of the wheels. The red trace on the left represents the total network activity (points depict evoked activity); the blue numbers in front of vehicle's “eyes” show distances (in cm) from the right and left sensed objects; the Edit distance of the evoked recruitment orders, from a predefined internal representation of the Right and Left objects, is shown in red numbers. Top left: time in seconds.
(3.4 MB MOV)
