# Supplementary figures and images for: Order-Based Representation in Random Networks of Cortical Neurons
Source: PLoS Comput Biol. 2008 Nov 21;4(11):e1000228. doi: 10.1371/journal.pcbi.1000228 (PMC2580731; doi:10.1371/journal.pcbi.1000228)

Duration of stimulation series (Sec)

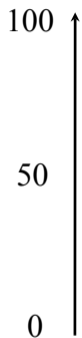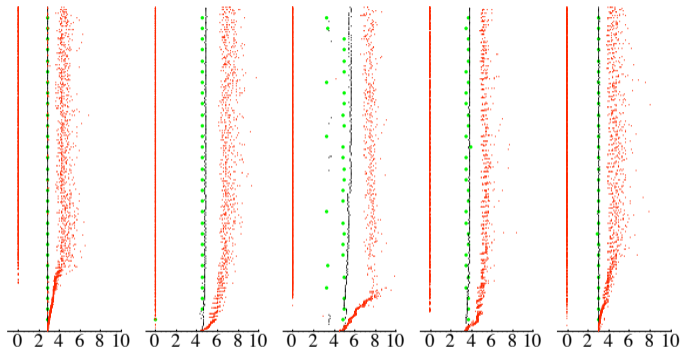

Post-stimulus time (mSec)

Supplement: Figure S1 — Dynamics of first spike latencies in pharmacologically isolated neurons. Excitatory synaptic transmission is blocked by application of 50 µM APV and 10 µM CNQX. As a result, all spontaneous activity and stimulus evoked reverberating responses were completely abolished. The network was then stimulated through all 60 electrodes sequentially to locate stimulation sites that effectively evoke direct, electrically induced action potentials. After selection of stimulating electrodes each network was stimulated by a short pulse (400 µSec) for 100 seconds (ordinate) at three different frequencies (0.3, 3 and 30/Sec; Green, Black and Red, respectively) in a shuffled manner. Five minutes were allowed for recovery between each stimulation series. Examples from five neurons are shown. The abscissa shows time post stimulus, in mSec. Failures to respond are plotted at time 0. Under these experimental conditions, neuronal responses to above-threshold stimuli reflect direct generation of action potentials in neurons that are nearby stimulating electrodes, free from the dynamics of synaptic transmission. At frequencies of 3/Sec or below, neurons are very reliable in their responses to directly delivered stimuli. At the higher frequency regimen, response jitter develops gradually over several seconds. (4.6 MB PDF) [file pcbi.1000228.s001.pdf]

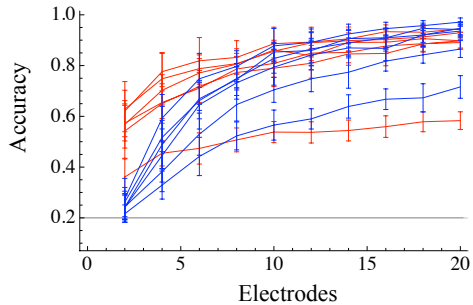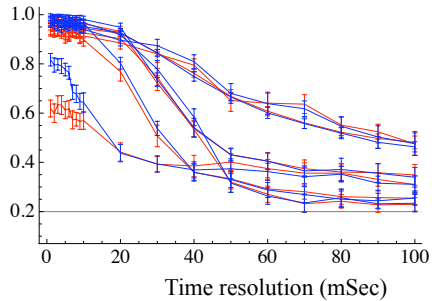

Supplement: Figure S3 — Examples of five-sources classification task. Results from six different networks that were challenged with a five-sources classification task. While variance in the sensitivities of different networks to both the number of sampled neurons and temporal resolution is apparent, the overall picture is fairly robust. Left: Sensitivity of classification accuracy to the number of sampled electrodes was measured in a five-stimulation sites classification task. Horizontal line depicts chance accuracy level. Support vector machine (SVM) algorithm with a Gaussian Radial Based function kernel was applied to vectors of training sets (see methods). The resulting classifiers were then validated using test sets vectors. Mean test accuracy and standard deviation are calculated from 10 random combinations of electrodes per given sample size from all analyzed electrodes, using a 10-fold cross validation procedure. Classification accuracy of first spike latency is depicted Red; that of recruitment order is depicted Blue. Right: Sensitivity of classification accuracy to to the temporal resolution of sampled latencies to first spike times was measured in a five-stimulation sites classification task. Horizontal line depicts chance accuracy level. Support vector machine (SVM) algorithm was applied (see methods and caption of Figure 7). Mean test accuracy and standard deviation are calculated from a 30-fold cross validation procedure per given time resolution. Classification accuracy of first spike latency is depicted Red; that of recruitment order is depicted Blue; adjacent Red-Blue curves result from the same experiment (network). (4.6 MB PDF) [file pcbi.1000228.s003.pdf]
