# Supplementary material for: Order-Based Representation in Random Networks of Cortical Neurons
Source: PLoS Comput Biol. 2008 Nov 21;4(11):e1000228. doi: 10.1371/journal.pcbi.1000228 (PMC2580731; doi:10.1371/journal.pcbi.1000228)
Supplement: Figure S2 — Pair-order probability matrices. More examples of pair-order probability matrices, similar to those shown in Figure 4A. Each boxed set of matrices comes from a different network. Each of the matrices within a box is constructed from responses to a different stimulation source (networks that were exposed to two and three input sources are shown). Numbers to the left of each box depict preparation (network) identity. All cases show ordered recruitment; in most cases matrices of different stimulation sources (within each box) look different. Indeed, the statistical analyses shown in Figures 6, 7, 8 and Supplementary Item 3 clearly indicate that these differences are significant and allow for rapid and reliable classification of inputs by use of unsupervised as well as supervised classifiers (15 networks tested altogether). Note: Neurons that are less “committed” to their average rank appear as horizontal (and vertical) smeared lines throughout the matrix. (4.6 MB PDF) [file pcbi.1000228.s002.pdf]

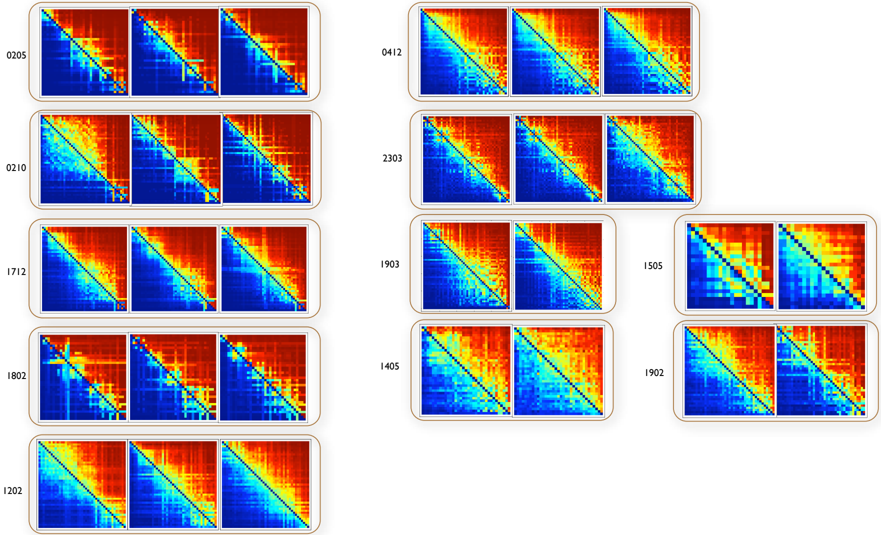
0205
0210
1712
1802
1202
0412
2303
1903
1405
1505
1902
